# Prevalence of Empyema or Mucocele or Other Histological Diagnoses in Patients Undergoing Cholecystectomy With Diagnosis of Chronic Cholecystitis

**DOI:** 10.7759/cureus.23773

**Published:** 2022-04-03

**Authors:** Munira Murtaza Khomusi, Sughra Parveen, Mazhar Iqbal, Tanweer Ahmed, Razia Husain, Uzma Shamim Seth, Jehangir Ali Soomro, Zainab Sariyah Khan

**Affiliations:** 1 Surgical Ward 1, Jinnah Postgraduate Medical Centre, Karachi, PAK; 2 Surgical Ward 2, Jinnah Postgraduate Medical Centre, Karachi, PAK; 3 Department of Anesthesiology, Bahawalpur Medical and Dental College, Bahawalpur, PAK; 4 Surgery, Government Hospital Ghaziabad, Lahore, PAK

**Keywords:** strawberry gallbladder, cholelithiasis, mucocele, empyema, chronic cholecystitis

## Abstract

Background

Chronic cholecystitis is inflammation of the gall bladder usually caused by stones. The aim was to find out the prevalence of mucocele and empyema in chronic cholecystitis using cholecystectomy findings and histopathological reports.

Methodology

This was a cross-sectional observational study conducted in Surgical Ward 1, Jinnah Postgraduate Medical Center, Karachi from December 2019 to December 2021 for two years. Patients above 12 years of age diagnosed with chronic cholecystitis with cholelithiasis on clinical examination and investigations were included. Patients who were diagnosed with acute cholecystitis, mucocele, or empyema on clinical examination and ultrasonography were excluded from the study. Laparoscopic cholecystectomy was done and operative findings were noted. Gall bladder specimens were examined for mucocele and empyema and were sent for histopathology. Results were recorded and analyzed.

Results

There were 241 patients diagnosed with chronic cholecystitis with cholelithiasis on clinical examination and investigations. On examination, tenderness in the right hypochondrium was absent in all patients. Chronic cholecystitis was proved on histopathology in 231 patients (95.85%). Other findings diagnosed on peroperative findings and histopathology reports were strawberry gall bladder (2.41%), empyema (0.83%), mucocele (0.41%), and polyp (0.41%). Two hundred eight patients were female (86.31%), 33 were male(13.69%). The male to female ratio was 1:6.43. The average age was 31 years.

Conclusion

Inflammation and fibrosis of the gallbladder around Calot’s triangle increase the chances of vascular and common bile duct injury. In such cases, cholecystectomy can become difficult. It was concluded that empyema, mucocele, and strawberry gall bladder could be found in chronic cholecystitis, and cholecystectomy becomes difficult in such cases.

## Introduction

Chronic cholecystitis is inflammation of the gall bladder resulting in its dysfunction. It presents with pain in the right hypochondrium radiating to the back and tip of the shoulder. On examination, the patient is vitally stable and mildly tender on deep palpation of the abdomen. In these patients, liver function tests and complete blood counts are usually normal. Ultrasound reveals cholelithiasis and thick-walled gall bladder. It can also be acalculous cholecystitis [1].

Most patients present in the fourth decade of life. 84.87% can present with chronic cholecystitis and 2.21% carcinoma can be present as an incidental finding. These carcinomas are usually asymptomatic, therefore, every cholecystectomy should be sent for histopathological reporting [2]. Sometimes incidental finding of polyp of the gall bladder is found. Treatment of polyp is cholecystectomy if the size is more than 10 mm. Polyps can also present with symptoms of chronic cholecystitis [3]. Chronic cholecystitis sometimes has adenomyomatosis or cholesterolosis as well. In this case, there is a thickening of mucosa of the gall bladder and smooth muscle hypertrophy. The submucosa is also invaded by cholesterol. It is also known as strawberry gall bladder [4].

Sometimes in chronic cholecystitis, we can find diverticulosis. Usually, it is very rare and left-sided gall bladder can be rarely found as well (0.04 to 1.1%) [5]. Mucocele of the gallbladder can be found incidentally during cholecystectomy. In mucocele, mostly gall bladder is palpable but it can be missed by the ultrasonologist and identified during cholecystectomy. Rupture of the gall bladder can occur, leading to peritonitis [6]. Empyema can present with symptoms of acute cholecystitis or sometimes as chronic cholecystitis. Its management is emergency cholecystectomy. In cholecystectomy due to empyema, the frequency of complications is increased [7]. Sometimes a fibrosed Calot’s triangle is found and cholecystectomy is difficult. In such cases, we have to do subtotal cholecystectomy which can avoid the complications of surgery. Subtotal cholecystectomy is lifesaving in these difficult cases [8].

The rationale of this study was to report the unusual findings in chronic cholecystitis which were missed on the diagnosis after physical examination and investigations viz. ultrasound, liver function tests, and complete blood count. These unusual findings sometimes produce difficulties for the surgeon. If these are found then cholecystectomy can become difficult. A surgeon should therefore be aware of these. We wanted to know the exact percentage of these pathologies in tertiary-care centers, so as to contribute knowledge to the other surgeons working in secondary centers. The objective of this study was to find out the prevalence of mucocele or empyema in chronic cholecystitis using cholecystectomy findings and histopathological reports.

## Materials and methods

This observational cross-sectional study was conducted in Surgical Ward 1, Jinnah Postgraduate Medical Center from December 2019 to December 2021 for two years. After approval was taken from Ethical Review Board NO.F.2-81/2019-GENL/51225/JPMC, dated 15 June 2019, data collection was commenced.

Two hundred and forty-one patients were enrolled in this study; patients were above 12 years of age. Patients with signs and symptoms of chronic cholecystitis like pain in right hypochondrium radiating to back and shoulder and on examination, mild tenderness on deep palpation were provisionally diagnosed as chronic cholecystitis in the outpatient department (OPD). Serum liver function tests, urea, creatinine, electrolytes, complete blood count, hepatitis B, hepatitis C, and ultrasonography of the abdomen were carried out in OPD. Acute conditions like deranged liver function tests, ultrasound findings of polyps or mucocele were excluded. Patients diagnosed on clinical examination with acute cholecystitis were excluded. Patients with gallbladder mass detected on ultrasonography were also excluded. Patients who had dilated common bile duct and jaundice due to stones were excluded. Patients diagnosed with chronic cholecystitis with cholelithiasis on examination and investigations were admitted to the ward for laparoscopic cholecystectomy. Laparoscopic cholecystectomy was done and, if needed, converted to open cholecystectomy especially when there was a difficult Calotʼs triangle or bleeding or ruptured empyema of the gallbladder was found incidentally. Gallbladder specimens were examined for rare findings like empyema, mucocele, or polyps. Specimens were sent for histopathological report and findings were recorded as mucocele, empyema, polyp, strawberry gallbladder or incidental gallbladder carcinoma, or any other pathology. Results were narrated as a frequency of rare pathologies of the gallbladder in chronic cholecystitis.

## Results

Two hundred and forty-one patients with chronic cholecystitis were included in the study. The average age was 31±8 years. Examinations of all patients who had no signs and symptoms of acute cholecystitis were done. Tenderness in the right hypochondrium was absent. The liver function test and total leukocyte count in all cases were normal (100%). Thirty-three were male (13.69%), 208 were female (86.31%), the male to female ratio was 1:6.30. On laparoscopic cholecystectomy, we found empyema and mucocele (1.24%) and strawberry gallbladder (98.26%). Histopathological diagnoses were mucocele, empyema, polyp, strawberry gallbladder, and carcinoma. Mainly diagnosis was chronic cholecystitis with cholelithiasis but incidentally, other findings were noted shown in Table 1.

**Table 1:** Histopathological Findings of Chronic Cholecystitis Specimens n = mean number of patients

Diagnosis	No. of patients n=241	Percentage
Chronic (follicular) cholecystitis	231/241	95.85
Strawberry gallbladder	6/241	2.49
Empyema	2/241	0.83
Mucocele	1/241	0.41
Polyp	1/241	0.41
Adenocarcinoma	0/241	0

## Discussion

Chronic cholecystitis is usually due to cholelithiasis but incidentally, other pathologies are found. Chronic cholecystitis is also a risk factor for carcinoma. In a study conducted in New Delhi, 0.2% cases of adenocarcinoma were found incidentally. It causes carcinoma as it causes irritation of gallbladder mucosa which invokes metaplastic and dysplastic transformation. The mean ages usually found are 45 to 77 years [9]. But in the study, adenocarcinoma was not found and mean age of 31 years was found.

Male to female ratio of 1:6.30 was found. Chronic cholecystitis mainly involves females; average age of 56 years was found in a study conducted in Istanbul [10]. In this study, average age of 31 years and predominantly females were found. In chronic cholecystitis, the patient has pain in the right hypochondrium only, but signs are less common like tenderness. In chronic cholecystitis, we usually do planned cholecystectomy [11]. We included the same type of cases in this study but during surgery incidentally, we found mucocele (0.41%) and empyema (0.83%). There should be tenderness present in these cases, however, we did not find any tenderness; these were diagnosed during surgery. In these patients, hospital stay was increased and antibiotics had to be given for a longer duration. Wound infection also occurred in patients of empyema.

Mucocele is usually caused by mucus filled in the gallbladder due to obstruction of the cystic duct with stone. The patient can present with acute cholecystitis, usually on examination gall bladder is distended and palpable. But in this study, we did not find palpable gall bladder. Liver function tests can also be deranged in these patients; however, in patients in this study liver function tests were normal. In mucocele after cholecystectomy, when gallbladder is opened, clear mucus is found [12]. In this study, we did not find any signs and symptoms of acute cholecystitis in cases of mucocele. It was only discovered during surgery. The gallbladder was distended and filled with mucus. The finding of mucocele was confirmed on histopathology. Gallbladder was not palpable in this study as compared to the abovementioned study. Cholecystectomy becomes difficult due to impacted stone at cystic duct. In such cases, the stone has to be pushed back into the gallbladder during surgery so that clips can be applied. Cholecystectomy becomes difficult incidentally; surgeons should be mentally prepared for such cases. The incidence of bile duct injury also increases when a stone is impacted in the cystic duct.

Empyema of gallbladder is a deadly complication of cholelithiasis. It usually has signs and symptoms of acute cholecystitis. Clinical findings of fever, tachycardia, and tenderness are usually present, but in our cases these were absent. It usually presents at 51.12 years of age. The Triangle of Calot’s is usually distorted and expertise should be available to operate these cases [13]. Empyema causes septicemia in older age groups and is fatal. But in this study, we found empyema (0.83%) which was initially diagnosed as chronic cholecystitis and was missed on clinical examination and investigations. We have to convert these cases from laparoscopic to open surgery. Most probably signs and symptoms were absent due to the judicious use of antibiotics by general physicians. In these patients, hospital stay was increased, wound infection occurred, and recovery was delayed.

Adenomyomatosis is a benign condition with hypertrophy of mucosa. It is also called strawberry gallbladder. It is usually 1 to 9% symptomatic adenomyomatosis; in these cases cholecystectomy is indicated. It presents with abdominal pain [14]. In this study, patients who presented had symptoms of chronic cholecystitis and after histopathology report revealed adenomyomatosis. In this study, 2.49% were strawberry gallbladder diagnosed incidentally on histopathology, and carcinoma was also excluded in these patients.

Gallbladder polyps greater than 1 cm are visible on ultrasonography. Sometimes they are symptomatic. Polyps are usually of two types injury-related and cholesterol-related. Cholesterolosis can also be found in these cases. Polyps are usually found in 3% of cholecystectomies [15]. But in this study, we found 0.41% incidentally, which presented as chronic cholecystitis. These polyps were less than 1 cm in size and missed on ultrasonography.

The limitation was the small sample size due to limited time of the study. If this study is prolonged for years, it can be slightly affected. We excluded children below 12 years of age, if we include these patients then incidence of polyps may be increased. Other congenital deformities like diverticulosis can also be found in children.

## Conclusions

Inflammation and fibrosis of gallbladder around Calot’s triangle increase the chances of vascular and common bile duct injury. In such cases, cholecystectomy can become difficult. It was concluded that empyema, mucocele, and strawberry gall bladder could be found in chronic cholecystitis and cholecystectomy becomes difficult in such cases.
